# Supplementary material for: Adiponectin and TNF-Alpha Differentially Mediate the Association Between Cystatin C and Oxidized LDL in Type 2 Diabetes Mellitus Patients
Source: Int J Mol Sci. 2025 Mar 25;26(7):3001. doi: 10.3390/ijms26073001 (PMC11988364; doi:10.3390/ijms26073001)
Supplement: Supplementary file 1 [file ijms-26-03001-s001.zip › ijms-3515384-supplementary.pdf]

Supplementary Table S1. Spearman correlation matrix of plasma and urinary Cystatin C, and Ox-LDL with other variables in T2DM patients

Variables		PlCys-C	UrCys-C	Age	Gender	HbA1c	Cre	Chol-t	LDL-c	HDL-c	TAG	Hs-CRP	BMI	Ox-LDL	NO	eGFR
PlCys-C	R	1	0.279*	0.065	-0.185	-0.062	0.215	-0.089	-0.121	-0.033	0.124	-0.046	-0.172	0.569***	0.042	-0.242
	P	.	0.036	0.631	0.168	0.653	0.112	0.554	0.423	0.829	0.419	0.762	0.201	0.001	0.758	0.072
UrCys-C	R	0.279*	1	-0.007	0.055	-0.058	-0.071	0.139	0.05	-0.182	0.031	-0.03	0.043	0.485***	-0.181	-0.077
	P	0.036	.	0.96	0.687	0.672	0.601	0.357	0.742	0.232	0.839	0.845	0.751	0.001	0.178	0.57
Age	R	0.065	-0.007	1	-0.076	0.044	0.186	-0.191	-0.241	0.042	-0.047	0.013	-0.063	0.082	-0.171	-0.384***
	P	0.631	0.96	.	0.574	0.751	0.17	0.204	0.107	0.783	0.759	0.933	0.642	0.543	0.203	0.003
Gender	R	-0.185	0.055	-0.076	1	-0.023	-0.429***	0.116	0.360*	-0.06	-0.292	0.053	0.167	0.064	-0.185	0.228
	P	0.168	0.687	0.574	.	0.865	0.001	0.443	0.014	0.697	0.051	0.727	0.214	0.635	0.167	0.091
HbA1c	R	-0.062	-0.058	0.044	-0.023	1	-0.003	0.062	0.1	0.178	0.063	0.147	0.204	-0.098	0.079	0.028
	P	0.653	0.672	0.751	0.865	.	0.981	0.683	0.51	0.242	0.681	0.34	0.135	0.478	0.567	0.843
Cre	R	0.215	-0.071	0.186	-0.429***	-0.003	1	-0.089	-0.113	-0.134	0.091	0.083	-0.166	0.099	0.247	-0.864***
	P	0.112	0.601	0.17	0.001	0.981	.	0.555	0.456	0.382	0.551	0.583	0.221	0.466	0.067	0.001
Chol-t	R	-0.089	0.139	-0.191	0.116	0.062	-0.089	1	0.661***	-0.253	0.297*	0.056	0.091	-0.048	0.04	0.165
	P	0.554	0.357	0.204	0.443	0.683	0.555	.	0.001	0.094	0.047	0.739	0.546	0.752	0.793	0.274
LDL-c	R	-0.121	0.05	-0.241	0.360*	0.1	-0.113	0.661***	1	-0.107	0.083	0.081	0.061	-0.032	0.002	0.173
	P	0.423	0.742	0.107	0.014	0.51	0.456	0.001	.	0.484	0.588	0.629	0.685	0.833	0.99	0.251
HDL-c	R	-0.033	-0.182	0.042	-0.06	0.178	-0.134	-0.253	-0.107	1	0.064	0.219	-0.236	-0.079	-0.144	0.165
	P	0.829	0.232	0.783	0.697	0.242	0.382	0.094	0.484	.	0.682	0.194	0.119	0.607	0.344	0.28
TAG	R	0.124	0.031	-0.047	-0.292	0.063	0.091	0.297*	0.083	0.064	1	0.165	0.109	-0.206	-0.085	0.011
	P	0.419	0.839	0.759	0.051	0.681	0.551	0.047	0.588	0.682	.	0.328	0.477	0.176	0.578	0.942
Hs-CRP	R	-0.046	-0.03	0.013	0.053	0.147	0.083	0.056	0.081	0.219	0.165	1	0.26	-0.149	-0.123	-0.141
	P	0.762	0.845	0.933	0.727	0.34	0.583	0.739	0.629	0.194	0.328	.	0.081	0.324	0.417	0.35
BMI	R	-0.172	0.043	-0.063	0.167	0.204	-0.166	0.091	0.061	-0.236	0.109	0.26	1	-0.119	0.002	0.107
	P	0.201	0.751	0.642	0.214	0.135	0.221	0.546	0.685	0.119	0.477	0.081	.	0.376	0.988	0.434
Ox-LDL	R	0.569**	0.485**	0.082	0.064	-0.098	0.099	-0.048	-0.032	-0.079	-0.206	-0.149	-0.119	1	0.152	-0.241
	P	0.001	0.001	0.543	0.635	0.478	0.466	0.752	0.833	0.607	0.176	0.324	0.376	.	0.258	0.074
NO	R	0.042	-0.181	-0.171	-0.185	0.079	0.247	0.04	0.002	-0.144	-0.085	-0.123	0.002	0.152	1	-0.123
	P	0.758	0.178	0.203	0.167	0.567	0.067	0.793	0.99	0.344	0.578	0.417	0.988	0.258	.	0.366
eGFR	R	-0.242	-0.077	-0.384**	0.228	0.028	-0.864**	0.165	0.173	0.165	0.011	-0.141	0.107	-0.241	-0.123	1
	P	0.072	0.57	0.003	0.091	0.843	0.001	0.274	0.251	0.28	0.942	0.35	0.434	0.074	0.366	.

Results are expressed as Spearman's Rho coefficient (R) and probability value (p) for 2-tailed Spearman correlation analysis. Significance: *, p≤0.05. Significance: *, p ≤ 0.05, **, p ≤ 0.001 and ***, p ≤ 0.0001. Abbreviations: SBP, Systolic blood pressure; DBP, Diastolic blood pressure; HbA1c, hemoglobin HbA1c; Cre, Creatinine; Chol-t, Total cholesterol; LDL-c, low-density lipoprotein cholesterol; HDL-c, high-density lipoprotein cholesterol; TAG, Triglycerides; BMI, Body mass index; Hs-CRP, high sensitivity C-reactive protein; eGFR, estimated glomerular filtration rate; ApoB, apolipoprotein B; Ox-LDL, oxidized low-density lipoprotein.

Supplementary Table S2.

Spearman correlation matrix of plasma and urinary Cystatin C, and Ox-LDL with metabolic hormones and cytokines in T2DM patients

Variables		PlCys-C	UrCys-C	Ox-LDL	ADP	NGF	IL-8	MCP1	IL-1b	GLP1	IL-6	INS	LEP	TNFα
PlCys-C	R	1.000	0.279*	0.569***	-0.346**	0.300*	0.024	0.104	0.051	0.194	0.397**	0.040	0.342**	0.663***
	P	.	.036	0.001	0.008	0.023	0.859	0.440	0.708	0.149	0.002	0.769	.009	.001
UrCys-C	R	0.279*	1.000	0.485***	-0.182	0.031	-0.084	-0.040	-0.203	0.214	0.164	0.177	0.223	0.333*
	P	0.036	.	0.001	0.175	0.821	.534	0.768	.129	0.110	0.222	0.189	0.095	0.011
Ox-LDL	R	0.569***	0.485***	1.000	-0.465***	0.175	.070	0.066	-0.136	0.225	0.340**	-0.148	0.353**	0.530***
	P	0.001	0.001	.	0.001	0.194	.603	0.626	0.312	0.092	0.010	0.273	0.007	0.001
ADP	R	-0.346**	-0.182	-0.465***	1.000	-0.021	0.128	0.152	-0.109	-0.143	-0.068	0.047	-0.179	-0.276*
	P	0.008	0.175	0.001	.	0.874	0.342	0.258	0.418	0.290	0.615	0.731	0.182	0.037
NGF	R	0.300*	0.031	0.175	-0.021	1.000	0.380**	0.027	0.355**	0.115	0.348**	0.109	0.283*	0.375**
	P	0.023	0.821	0.194	0.874	.	0.004	0.845	0.007	0.395	0.008	0.421	0.033	0.004
IL-8	R	0.024	-0.084	0.070	0.128	0.380**	1.000	0.149	0.427***	-0.061	0.265*	-0.072	-0.181	0.364**
	P	0.859	0.534	0.603	0.342	0.004	.	0.269	0.001	0.655	0.046	0.592	0.178	0.005
MCP1	R	0.104	-0.040	0.066	0.152	0.027	0.149	1.000	-0.098	0.095	0.183	-0.038	0.102	0.171
	P	0.440	0.768	0.626	0.258	0.845	0.269	.	0.469	0.484	0.174	0.778	0.452	0.204
IL-1b	R	0.051	-0.203	-0.136	-0.109	0.355**	0.427***	-0.098	1.000	-0.207	0.089	-0.034	-0.185	0.153
	P	0.708	0.129	0.312	0.418	0.007	0.001	0.469	.	0.123	0.513	0.804	0.168	0.256
GLP1	R	0.194	0.214	0.225	-0.143	0.115	-0.061	0.095	-0.207	1.000	0.008	0.316*	0.053	0.114
	P	0.149	0.110	0.092	0.290	0.395	0.655	0.484	0.123	.	0.954	0.016	0.698	0.398
IL-6	R	.397**	0.164	0.340**	-0.068	0.348**	0.265*	0.183	0.089	0.008	1.000	0.030	0.353**	0.529***
	P	0.002	0.222	0.010	0.615	0.008	0.046	0.174	0.513	0.954	.	0.824	0.007	0.001
INS	R	0.040	0.177	-0.148	0.047	0.109	-0.072	-0.038	-0.034	0.316*	0.030	1.000	0.204	0.005
	P	0.769	0.189	0.273	0.731	0.421	0.592	0.778	0.804	0.016	0.824	.	0.129	0.969
LEP	R	0.342**	0.223	0.353**	-0.179	0.283*	-0.181	0.102	-0.185	0.053	0.353**	0.204	1.000	0.237
	P	0.009	0.095	0.007	0.182	0.033	0.178	0.452	0.168	0.698	0.007	0.129	.	0.075
TNFα	R	00.663***	0.333*	0.530***	-0.276*	0.375**	0.364**	0.171	0.153	0.114	0.529***	0.005	0.237	1.000
	P	.001	0.011	0.001	0.037	0.004	0.005	0.204	0.256	0.398	0.001	0.969	0.075	.

Results are expressed as Spearman's Rho coefficient (R) and probability value (p) for 2-tailed Spearman correlation analysis. Significance: *, p≤0.05, **, p ≤ 0.001 and ***, p ≤ 0.0001. Abbreviations: ox-LDL, oxidized low-density lipoprotein; ADP, Adiponectin; NGF, nerve growth factor; IL-8, interleukin 8; MCP1, monocyte chemoattractant protein-1; IL-1b, interleukin 1b; GLP1, glucagon-like peptide 1; IL-6, interleukin 6; INS, Insulin; LEP, Leptin; TNFα, tumor necrosis factor alpha; Hs-CRP, High sensitivity C-reactive protein.
